# Four modifiable factors that mediate the effect of educational time on major depressive disorder risk: A network Mendelian randomization study

**DOI:** 10.1371/journal.pone.0288034

**Published:** 2023-07-12

**Authors:** Bangbei Wan, Yamei Wu, Ning Ma, Zhi Zhou, Weiying Lu

**Affiliations:** 1 Reproductive Medical Center, Hainan Women and Children’s Medical Center, Haikou, China; 2 Department of Urology, Central South University Xiangya School of Medicine Affiliated Haikou Hospital, Haikou, China; Yale University School of Medicine, UNITED STATES

## Abstract

**Background:**

Major depressive disorder (MDD) is a mental illness, which is a notable public health problem that aggravates the global economic burden. This study aimed to investigate the causal relationship between education and MDD risk and the contributions of effects mediated by four modifiable factors.

**Materials and methods:**

Instrumental variables were screened from several large-scale genome-wide association study (GWAS) data (years of schooling with 766,345 participants, MDD with 59,851 cases and 113,154 controls, neuroticism with 329,821 individuals, smoking behavior with 195,068 cases and 164,638 controls, body mass index [BMI] with 336,107 individuals, and household income with 397,751 individuals). The data were used to evaluate the association of the four modifiable factors (neuroticism, smoking behavior, BMI, and household income) that mediate the effect of education on MDD risk via Mendelian randomization (MR) analysis.

**Results:**

Each standard deviation increase in years of schooling could reduce the risk for MDD by 30.70%. Higher neuroticism and BMI were associated with a higher risk of MDD. Non-smoking status and increased household income were protective factors for MDD. Notably, the mediator neuroticism, BMI, smoking behavior, and household income explained 52.92%, 15.54%, 31.86%, and 81.30% of the effect of years of schooling on MDD risk, respectively.

**Conclusions:**

Longer years of schooling have a protective effect on MDD risk. Reasonable interventions to reduce neuroticism, BMI, smoking, and increasing household income are beneficial for MDD prevention. Our work provides new ideas for the development of prevention strategies for MDD.

## Introduction

Major depressive disorder (MDD) is a psychological illness with a high prevalence rate and a severe effect on the physical and mental health of people throughout the world [1, 2]. Studies indicate that the prevalence rate of MDD is rising annually. Based on a previous meta-analysis survey that includes 105 studies, the prevalence of MDD was 5.8% in women and 3.5% in men [3]. A recent observational meta-analysis that included 20 studies involving 18,953 individuals revealed that the global morbidity of MDD was approximately 13.3% in older people [1]. According to statistical data from an observational study, the proportion of adults with MDD increased by 12.9% between 2010 and 2018, from 15.5 million to 17.5 million people in the United States [4]. In addition, statistics from the Global Burden of Disease Study indicate that the annual disability-adjusted life rate of major depression due to bullying has increased by 26.60 percent globally, with 29.07 percent for women and 23.84 percent for men, from 1990 to 2019 [5]. Although the psychological and pharmacologic treatments of MDD have made much progress, the cure rate remains relatively low, and the recrudescence remains high [6]. Considering that MDD is a refractory disease, the early identification of risk factors and taking effective interventions are very beneficial for preventing MDD.

MDD is a complex multifactorial mental illness. Social, personal, educational, and familial factors are closely related to the incidence of MDD [6–9]. For example, the study of Freeman et al. [10] revealed a significant association between depression and socio-economic status (SES). In addition, an observational study reported that cigarette smoking is an unhealthy behavior and can increase the risk of depression [11]. Furthermore, according to the data from an observational study, people with high BMI are likely to develop MDD [12]. Notably, neuroticism is a personality trait and a recognized risk factor for depression [13]. Although these findings are helpful, considering the environmental confounding and the possibility of reverse causation, the assessment of causal effects in observational research is difficult.

Mendelian randomization (MR) investigation is a reliable epidemiological method to infer the causality between interesting exposure (s) and outcome (s) by using genetic variants as instrumental variables [14, 15]. In addition, network MR is a powerful approach for assessing mediation [16, 17]. Mediation analysis can improve etiological understanding and identify intermediate variables as potential targets for intervention when intervention exposure is difficult [16]. In the current work, we comprehensively investigated the role of four traits (neuroticism, body mass index [BMI], smoking behavior, and average total household income before tax) in mediating the causal effect of education on the risk of MDD by using network MR. The inverse variance weighted (IVW) method was adopted as the primary algorithm to estimate causal effect size. Based on the importance of years of schooling (education), neuroticism, BMI, smoking behavior, and average total household income before tax (income) in triggering MDD, further understanding of the possible mechanism was very helpful in mapping out public health policy for MDD prevention.

## Materials and methods

### Overall study design

**Fig 1:**
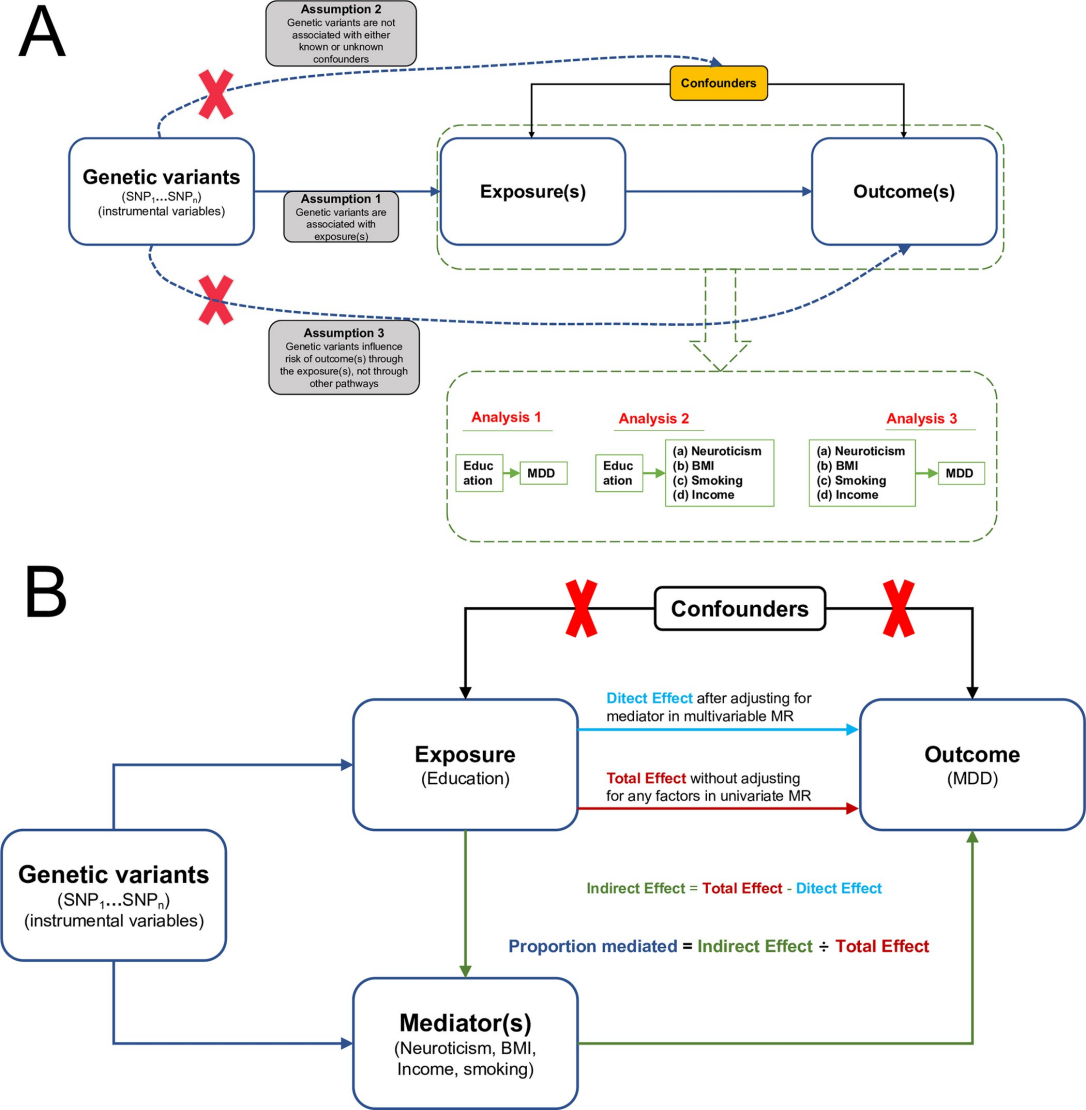
Schematic diagram of univariable and multivariable MR analyses. **(A) Univariable MR.** Fundamental assumptions for MR: (1) Relevance assumption: The genetic variants (instrumental variables) must be strongly correlated with exposure(s) (*P* < 5×10^−8^; *r*^2^ < 0.001 and distance >10,000 kb, the SNPs in pairwise linkage disequilibrium). (2) Independence assumption: no unmeasured confounders are involved in the correlations between genetic variants and outcome(s). (3) Exclusion restriction assumption: the genetic variants influence the outcome(s) only via exposure(s). **(B) Multivariable MR. MR =** Mendelian randomization; **SNP =** single nucleotide polymorphism; **MDD =** major depressive disorder; **BMI =** body mass index; **Education =** years of schooling; **Smoking =** smoking (ever *vs*. never); **Income =** average total household income before tax.

The overall study design schematic diagram of the MR is exhibited in Fig 1A and 1B. The summary statistical data for the exposure (s), mediator (s), and outcome (s)-related genome-wide association study (GWAS) were extracted from the IEU Open GWAS database (https://gwas.mrcieu.ac.uk/). The genetic variants strongly associated with exposure (s) and mediator (s) were used as the instrumental variables. The univariate IVW method was used to test the causality between exposure(s), mediator(s), and outcome(s). The multivariable IVW method was used to calculate the direct and indirect effect sizes from exposure (s) to the outcome. The proportion of mediating effect was generated using the indirect effect size divided by the total effect size [16].

### Ethical statement

Ethical approval and consent were not required as this study was based on publicly available data.

### Data sources

All GWAS summary-level data were obtained from a large-sample study of European populations. The education (years of schooling, standard deviation: 4.2 years) associated with data originated from a GWAS study with 766,345 individuals [18]. The neuroticism-related GWAS data was derived from 329,821 White British adult participants [19]. The BMI-related summary data involving 336,107 people were obtained from a GWAS analysis of the Neale lab. The income (average total household income before tax) associated with genetic data with 397,751 volunteers was obtained from a GWAS analysis of the UK Biobank. The smoking (ever *vs*. never)-related summary statistical data of 359,706 populations was derived from a GWAS analysis of the UK Biobank. The MDD-related summary-level data were extracted from a GWAS meta-analysis of 59,851 MDD cases and 113,154 controls [20]. Details of all data are shown in **S1 Table**.

### Statistical analysis

#### Univariable MR analysis

Network relationships among the exposure, mediators, and outcome were determined by performing univariable two-sample MR analyses by using the IVW method. A graphical summary of analyses is shown in Fig 1A. First, single nucleotide polymorphisms (SNPs) that are independently linked with years of schooling were selected as the instrumental variables to examine the causal relationship between exposure (education) and outcome (MDD) by using the IVW method. The instrumental variables were identified based on the following standards: (a) *P* < 5×10^−8^ as a genome-wide statistical significant threshold in the correlation between SNPs and exposure; (b) The parameter (*r*^2^ < 0.001 and clump window >10,000 kb, among SNPs) in pairwise linkage disequilibrium (LD) were deemed as the independent threshold of SNPs. In addition, the F statistic was used to evaluate the instrument strength in univariate MR [21]. An F statistic greater than 10 indicated the absence of instrument bias [22]. Next, the causal direction from exposure (education) to the mediators (neuroticism, BMI, smoking behavior, and income) was analyzed using univariable IVW regression. The standard for the selection of independent SNPs (instrumental variables) is described above. Finally, the causality between the per mediator and the outcome (MDD) was studied. The abovementioned method was employed.

The stability and dependability of the univariate MR analyses was verified by performing a battery of sensitivity analyses. First, MR Steiger test was used to examine the correctness of causal assumptions in the MR analyses. Second, the MR-Egger [23], Maximum likelihood [24], MR-pleiotropy residual sum outlier (MR-PRESSO) [25], and MR-robust adjusted profile score (MR-RAPS) [26] methods were employed to prove the consistency of causal hypothesis in IVW regression. Third, the statistical power of MR analyses was evaluated using an available online tool (https://shiny.cnsgenomics.com/mRnd/) [27]. A power greater than 80% was regarded as favorable evidence. Fourth, the IVW and MR-Egger models were used to estimate the heterogeneity of SNPs by using Cochran’s Q test [28]. A *P* value > 0.05 indicated no heterogeneity in the included instrumental variables. Hence, the influence of heterogeneity on the assessment of causal effects could be disregarded. In the presence of heterogeneity, the random-effects model was employed to determine the effect size [29, 30]. Sixth, the MR-Egger regression was used to inspect potential pleiotropy. The MR-PRESSO, MR-Egger, and IVW approaches were used to identify and remove potential outliers that can cause underlying pleiotropy. Finally, the leave-one-out permutation method was used to examine whether an existing single SNP can alter the pooled effect of IVW.

#### Multivariable MR analysis

Multivariable MR analysis was carried out to elucidate the causal effect of years of schooling on the risk of MDD mediated by neuroticism, BMI, smoking behavior, and income. A graphical summary of analyses is shown in Fig 1B. First, education and a single mediator in turn (including neuroticism, BMI, smoking behavior, and income) were included for multivariate MR to assess the direct and indirect effect (mediated by per mediator) of education influence on MDD. Next, education and the four mediators were included in multivariate MR to calculate the direct and indirect effect (mediated by the four mediators) of education influence on MDD. The proportion mediated (PM) was calculated using the indirect effect divided by the total effect, where the total effect originated from the univariable MR analysis [16].

All statistical analyses in MR were implemented using the TwoSampleMR (version 0.5.6) and MR-PRESSO packages in R (version 4.1.2).

## Results

### Network relationship among education, MDD, and the four mediators in univariable MR

**Fig 2:**
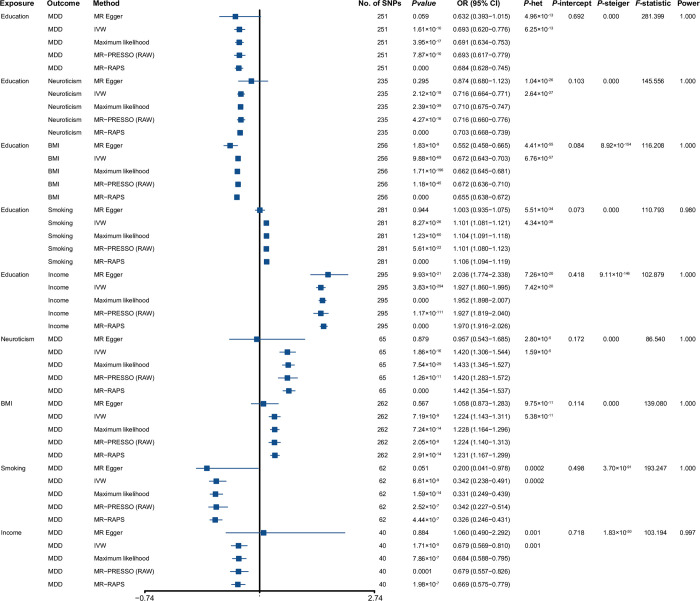
The forest plot denotes univariable MR analysis. **MR =** Mendelian randomization; **OR =** odds ratio; **CI =** confidence intervals; ***P*-het =** *P* value for heterogeneity using Cochran Q test; ***P*-intercept** = *P* value for MR-Egger intercept; ***P*-Steiger =** *P* value for MR-Steiger test; **IVW =** inverse variance weighted; **MR-PRESSO** = Mendelian randomization-pleiotropy residual sum outlier; **MR-RAPS =** robust adjusted profile score; **SNP =** single-nucleotide polymorphism; **MDD =** major depressive disorder; **BMI =** body mass index; **Education =** years of schooling; **Smoking =** smoking (ever *vs*. never); **Income =** average total household income before tax.

First, the causality between education and MDD was analyzed using univariable MR. After removing the outliers, all 251 independent SNPs were included to estimate the causal relationship between education and MDD. The results showed that genetically determined longer education was correlated with a lower risk of MDD with an odds ratio (OR) of 0.693 (95% confidence interval [CI]: 0.620–0.776, *P* = 1.61 × 10^−10^; **Fig 2**).

Next, the causal relationship between education and the four mediators (neuroticism, BMI, smoking behavior, and income) was investigated using univariable IVW regression. After deleting outliers, 235, 256, 281, and 295 SNPs were included in IVW regression to clarify the causality between education and four mediators. Results show that education was negatively correlated with neuroticism (OR = 0.716 [95% CI: 0.664–0.771], *P* = 2.12 × 10^−18^) and BMI (OR = 0.672 [95% CI: 0.643–0.703], *P* = 9.88 × 10^−69^) and positively associated with non-smoking behavior (OR = 1.101 [95% CI: 1.081–1.121], *P* = 8.27 × 10^−26^) and income (OR = 1.927 [95% CI: 1.860–1.995], *P* = 2.83 × 10^−294^; **Fig 2**).

Finally, the causality between the four mediators and the risk of MDD were assessed using the univariable IVW method. After removing outliers, all 65, 262, 67, and 40 SNPs were independently associated with neuroticism, BMI, smoking behavior, and income, respectively. The SNPs were then included in the univariable IVW analysis to evaluate the causality between the four mediators and MDD risk. The results showed that neuroticism (OR = 1.420 [95% CI: 1.306–1.544], *P* = 1.86 × 10^−16^) and BMI (OR = 1.224 [95% CI: 1.143–1.311], *P* = 7.19 × 10^−9^) were positively correlated with the risk of MDD. Smoking behavior (OR = 0.342 [95% CI: 0.238–0.491], *P* = 6.61 × 10^−9^) and income (OR = 0.679 [95% CI: 0.569–0.810], *P* = 1.71 × 10^−5^) were negatively associated with the risk of MDD (**Fig 2**).

In the above univariable MR analyses, all F statistics were more than 10, and all power values were almost 100%, showing no weak-instrument bias and excellent reliability (**Fig 2**). All SNP information is displayed in **S2–S10 Tables**. In addition, all results before removing the outliers can be seen in **S11 Table**.

**Fig 3:**
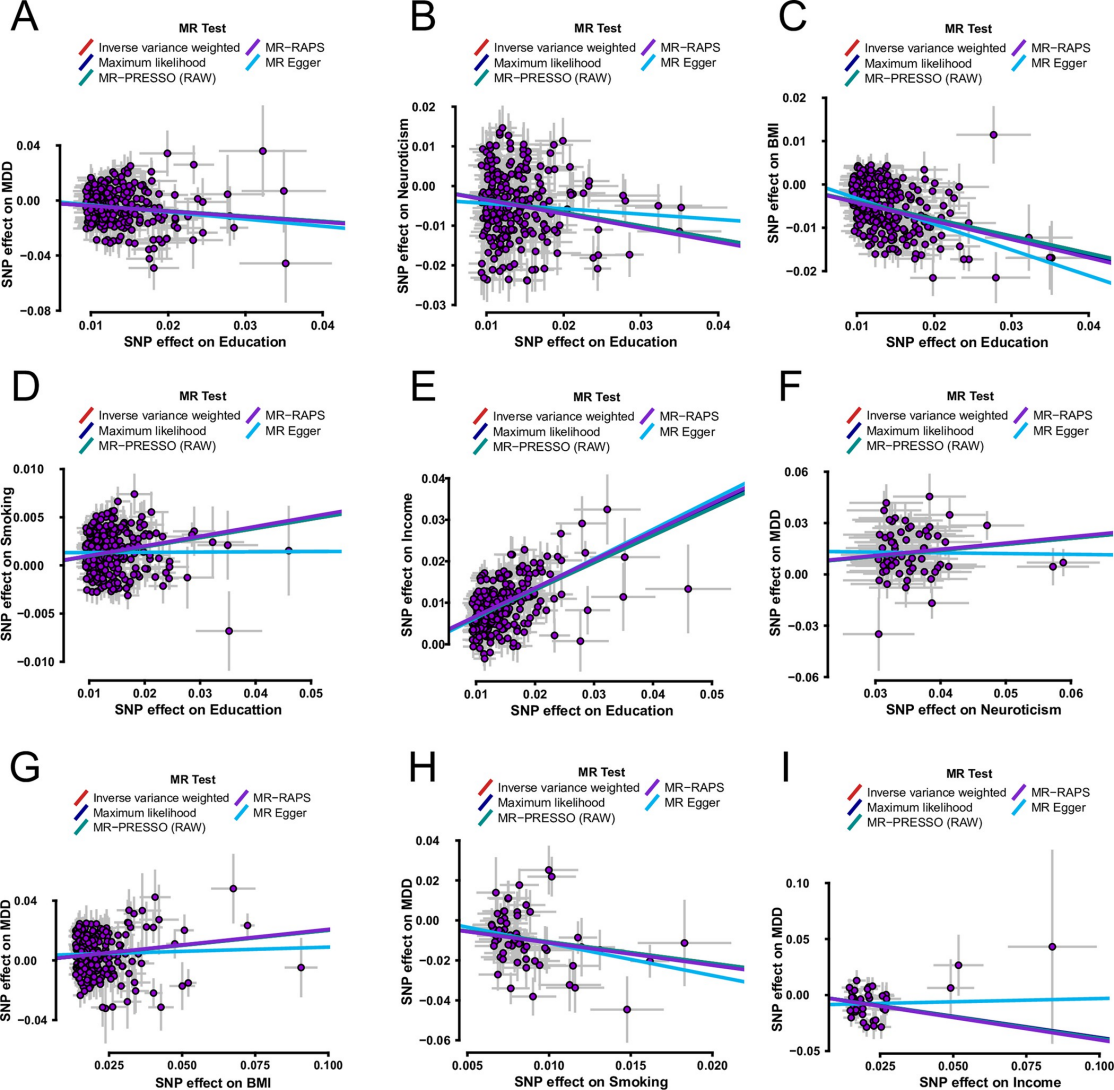
The scatter plots exhibit the IVW regression direction tested by four methods. **MR =** Mendelian randomization; **IVW =** inverse variance weighted; **MR-PRESSO =** Mendelian randomization-pleiotropy residual sum outlier; **MR-RAPS =** robust adjusted profile score; **SNP =** single-nucleotide polymorphism; **MDD =** major depressive disorder; **BMI =** body mass index; **Education =** years of schooling; **Smoking =** smoking (ever *vs*. never); **Income =** average total household income before tax.

A series of sensitivity analyses were used to assess the robustness and dependability of the above MR investigations. Four methods (MR-Egger, Maximum likelihood, MR-PRESSO, and MR-RAPS) displayed consistent direction with the IVW approach, suggesting that all causal assumptions were stable in the univariable MR analyses (**Fig 3** and **S1 Fig**). Second, the heterogeneity of SNPs in each MR analysis was investigated using Cochran’s Q statistic in the IVW and the MR-Egger methods. The results showed specific heterogeneity among SNPs (all *P*_-het_ < 0.05) per MR analysis (**Fig 2**). Accordingly, the random-effects model was used to directly estimate the aforementioned MR effect size. The heterogeneities were generated using Mendel’s law of independent assortment rather than by existing pleiotropy [31, 32]. Third, the statistical result of the MR-Egger regressions showed no directional pleiotropy in the MR analyses (all *P*_-intercept_ > 0.05; **Fig 2**). Fourth, the reduplicative leave-one-out test was used to inspect whether a single SNP observably transformed the combined effect of the IVW method. The results displayed that no single SNP markedly altered the combined effect of IVW (**S12–S20 Tables**). Finally, all causal directions were verified using the MR Steiger test. The results showed that all causal hypotheses were correct (all *P* < 0.000; **Fig 2**).

#### Multivariable MR analysis

**Fig 4:**
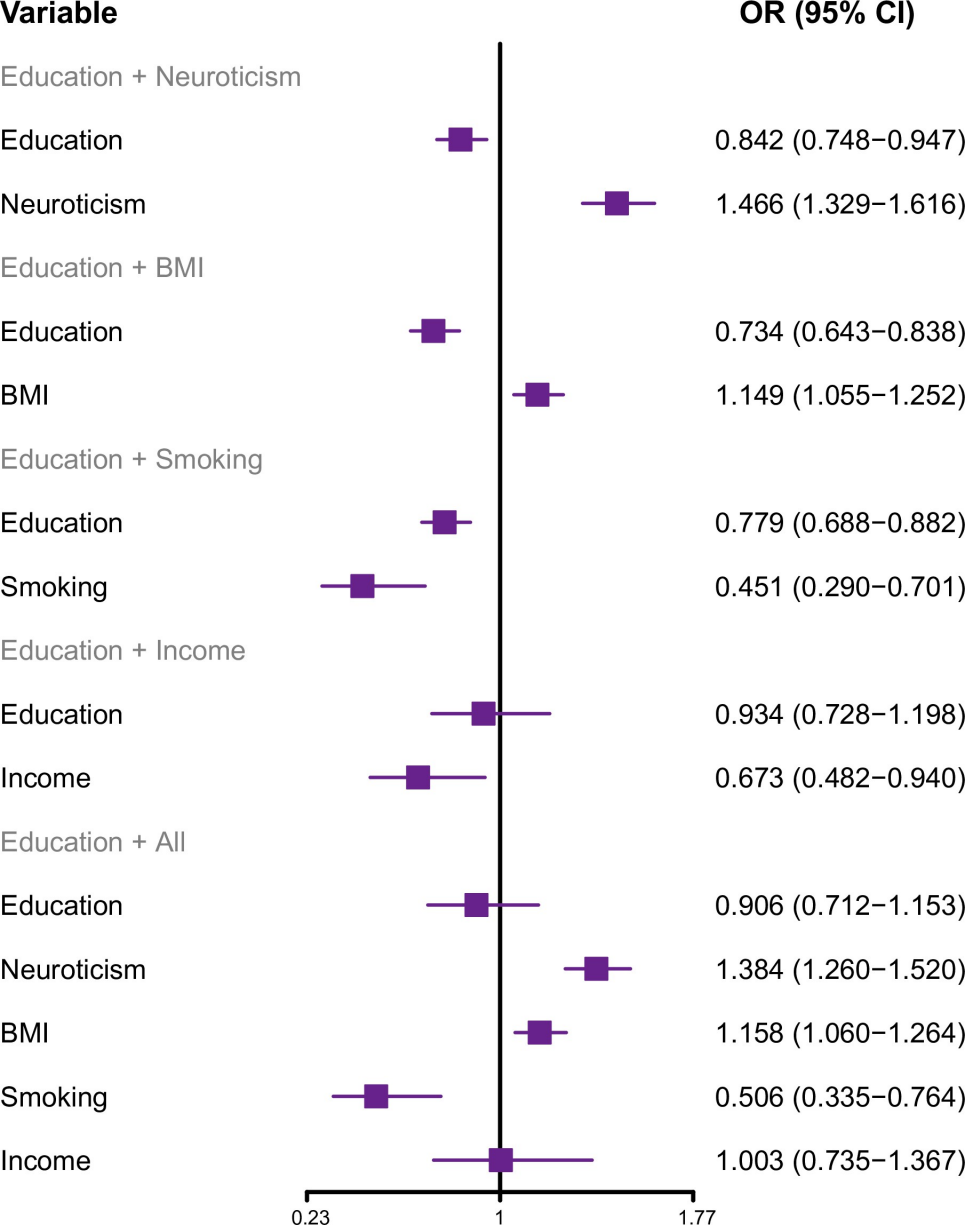
The forest plot exhibits multivariable MR analysis. **OR =** odds ratio; **CI =** confidence intervals; **MDD =** major depressive disorder; **BMI =** body mass index; **Education =** years of schooling; **Smoking =** smoking (ever *vs*. never); **Income =** average total household income before tax.

To clarify the potential mechanism of education influence on MDD mediated by the four mediators, we performed multivariable MR analyses. The PM per individual mediator or the combination of all mediators was analyzed (s). After correcting for neuroticism, the direct effect of education on MDD had an OR of 0.842 (95% CI: 0.748–0.947; **Fig 4**). The PM of neuroticism was 52.92%. When controlling for BMI, the result from multivariable MR showed that the direct effect of education on MDD had an OR of 0.734 (95% CI: 0.643–0.838; **Fig 4**). The PM of BMI was 15.54%. When correcting for smoking behavior (ever *vs*. never), the result of multivariable MR displayed the direct effect of education on MDD with an OR of 0.779 (95% CI: 0.688–0.882; **Fig 4**). The PM of smoking behavior was 31.86%. When rectifying income, the result from multivariable MR showed the direct effect of education on MDD with an OR of 0.934 (95% CI: 0.728–1.198; **Fig 4**). The PM of income was 81.30%. All the above results suggested that higher education levels can attenuate the risk of MDD by mediating the four factors at different levels. After controlling for all four mediators, the direct effect of education on MDD had an OR of 0.906 (95% CI: 0.712–1.153; **Fig 4**). The PM of the four mediators was 73.17%.

## Discussion

In the current work, large-scale GWAS data were used to investigate the network relationship among education, neuroticism, BMI, smoking behavior, income, and risk of MDD. Genetically predicted higher education levels can reduce the risk of MDD by regulating neuroticism, BMI, smoking behavior, and income.

The persistent prevalence of MDD has been a severe public health problem and has aggravated the global economic burden [33]. The development of effective preventive strategies for MDD is essential for resolving the issue, and the potential causes of MDD needs to be acknowledged to develop these policies. Studies indicate that education plays a vital role in the occurrence and progression of depression. Interestingly, in adult women, longer time spent on education (>16 years) was associated with decreased incidence of depression than limited education (12 years) with an OR of 0.61 [34]. Furthermore, a broad worldwide sample survey discovered a relationship between the number of years spent in education and decreased incidence of depression [10]. A secondary analysis conducted by Wickersham et al. also suggested that higher educational attainment was associated with a lower risk of depression [35]. Although the above observational findings revealed the association between education and depression, the causal inference was necessary because of environmental confounding and the possibility of reverse causation. Accordingly, MR analysis was used to make up for the defect. In line with previous observational studies, our findings showed a negative causal association between years of schooling and the risk of MDD with OR of 0.693.

Neuroticism is a character trait with sensitivity and mood swings and is a risk factor for depression [19, 36, 37]. Observational and MR investigations revealed that neuroticism was positively correlated with the risk of MDD [38–40]. The negative association between the years of schooling and neuroticism was disclosed in the observational data [41]. However, the causal relationship between schooling years and neuroticism remains unclear. Whether years of schooling affects MDD by regulating neuroticism still need to be proved. In the present work, longer years of schooling were correlated with a lower risk of neuroticism (OR: 0.716, 95% CI: 0.664–0.771). The effect of years of schooling on MDD was mediated by neuroticism, and the PM was 52.92%.

BMI is a primary measure of obesity and is associated with the risk of depression. Based on a meta-analysis that included 15 studies involving 58,745 individuals, obesity can increase the risk of depression, but the occurrence of depression can promote the risk of obesity [42]. A meta-analysis with a large sample size also reported that obesity was positively associated with the risk of depression with a RR (risk ratio) of 1.18 (95% CI: 1.04–1.35), while depression increased the risk of obesity (RR = 1.37, 95% CI: 1.17–1.48) [43]. Observational studies suggested a bidirectional association between obesity and depression. In the present work, a genetically predicted 1-SD increase in BMI was correlated with a high risk of MDD with an OR of 1.224 (95% CI: 1.143–1.311). Higher educational attainment reduced the risk of obesity in the observational trial [44]. An analogous phenomenon was also observed in a recent investigation that people with high educational attainment focus more on health maintenance [45]. Likewise, our results supported the causality between years of schooling and BMI. Based on the above evidence, BMI could mediate the effect of years of schooling on MDD, and the PM was 15.54%.

Tobacco contains many harmful ingredients and is related to the risk of diseases [46]. A bidirectional association was observed between smoking behavior and depression, but the causality remains unexplained [47]. Our work indicated a positive causal relationship between smoking behavior and depression. Individuals who never smoked have a lower risk of MDD than those with smoking behavior (OR = 0.342, 95% CI: 0.238–0.491). A correlation between education and smoking behavior was reported in the observational study [48]. People with higher educational attainment are less likely to smoke [49, 50]. Our MR study also suggested that genetically predicted prolonging years of schooling can reduce the occurrence of smoking behavior. Furthermore, our further multivariable MR showed that years of schooling could decrease the risk of MDD by regulating smoking behavior, and the PM was 31.86%.

Household income plays a vital role in depression [51]. The populations in the family with low income have a higher depressive risk than those in high-income families [51]. The observational data from adolescents suggested that the depressive risk of the adolescents in lower parental income families was twice as much as those in higher parental income families [52]. Our univariable MR analysis indicated that a genetically predicted 1-SD increase in average total household income before tax was correlated with decreasing risk for MDD. Education is among the crucial influencing factors for income [53, 54]. Based on univariable MR, the effect of years of schooling on average total household income before tax was a correct causal direction. Further multivariable MR indicated that the average total household income before tax is among the mediators in longer years of schooling attenuated the risk of MDD, and the PM was 81.30%. This finding supports that average total household income before tax may be among the most important factors that mediate the effect of years of schooling on MDD. Considering that the effect of the four mediators on the risk of MDD differed, the amalgamative PM of the four mediators was evaluated. When four mediators were included together, the PM remained high at 73.17%. Based on the above evidence, increasing educational attainment may be a very beneficial strategy to attenuate the risk of MDD for people with differences in neuroticism, BMI, smoking behavior, and average total household income before tax. Besides, changing the four mediators may also be an effective policy to interrupt the high risk of MDD caused by lower educational levels.

Our work had several limitations. First, the genetically determined effect of years of schooling on MDD is mediated by the four factors (neuroticism, BMI, smoking behavior, and average total household income before tax) in a lifetime. Therefore, provisional clinical intervention cannot eliminate the risk of MDD. Second, although our work primarily revealed the mechanism of education on the effect on the risk of MDD, a small possible effect remains unexplained, and whether existing bidirectional causality between years of education, the four factors, and MDD remains unclear. Third, owing to the limited data, the effect of years of schooling on MDD was not explained by data stratification according to different ages and gender. Fourth, the results of MR analyses were obtained from European populations, and whether they can be popularized to non-European ancestry still need to be validated further. Finally, the potential biological mechanism of the effect of educational time on major depressive disorder risk mediated by the four modifiable factors is still unclear. Hence, the more molecular experiment is necessary to validate the finding of this study.

## Conclusion

In conclusion, this work used summarized genetic data to investigate the complex causal relationship among education, neuroticism, BMI, smoking behavior, average total household income before tax, and MDD risk. Our work revealed that years of schooling regulate the risk of MDD primarily mediated by neuroticism, BMI, smoking behavior, and average total household income before tax. Reasonably improving educational time, neuroticism, BMI, smoking behavior, and household income are beneficial for MDD prevention. These findings provide new ideas for the development of prevention strategies for MDD.

## Supporting information

S1 Fig Density plots show that the five methods estimate the effect of SNPs on the outcome of interest. (A) Effect of education-related SNPs on the risk of MDD. (B) Effect of education-related SNPs on neuroticism. (C) Effect of education-related SNPs on BMI. (D) Effect of education-related SNPs on smoking. (E) Effect of education-related SNPs on income. (F) Effect of neuroticism-related SNPs on the risk of MDD. (G) Effect of BMI-related SNPs on the risk of MDD. (H) Effect of smoking-related SNPs on the risk of MDD. (I) Effect of income-related SNPs on the risk of MDD. **MDD =** major depressive disorder; **BMI =** body mass index; **Education =** years of schooling; **Smoking =** smoking (ever *vs*. never); **Income =** average total household income before tax; **MR =** Mendelian randomization; **SNP =** single nucleotide polymorphism; **IVW =** inverse-variance-weighted; **MR-PRESSO =** MR-pleiotropy residual sum outlier; **MR-RAPS =** MR-robust adjusted profile score.
(TIF)

S1 Table Details of GWAS summary data. (XLSX)

S2 Table The detailed information on single-nucleotide polymorphisms for estimating the causal association between years of schooling and major depressive disorder. (XLSX)

S3 Table The detailed information on single-nucleotide polymorphisms for estimating the causal association between years of schooling and neuroticism. (XLSX)

S4 Table The detailed information on single-nucleotide polymorphisms for estimating the causal association between years of schooling and BMI. (XLSX)

S5 Table The detailed information on single-nucleotide polymorphisms for estimating the causal association between years of schooling and average total household income before tax. (XLSX)

S6 Table The detailed information on single-nucleotide polymorphisms for estimating the causal association between years of schooling and smoking. (XLSX)

S7 Table The detailed information on single-nucleotide polymorphisms for estimating the causal association between neuroticism and major depressive disorder. (XLSX)

S8 Table The detailed information on single-nucleotide polymorphisms for estimating the causal association between body mass index and major depressive disorder. (XLSX)

S9 Table The detailed information on single-nucleotide polymorphisms for estimating the causal association between smoking and major depressive disorder. (XLSX)

S10 Table The detailed information on single-nucleotide polymorphisms for estimating the causal association between income and major depressive disorder. (XLSX)

S11 Table Summary of univariable MR results before removing the outliers. (XLSX)

S12 Table MR leave-one-out analysis of the causal effect of years of schooling on major depressive disorder. (XLSX)

S13 Table MR leave-one-out analysis of the causal effect of years of schooling on neuroticism. (XLSX)

S14 Table MR leave-one-out analysis of the causal effect of years of schooling on body mass index. (XLSX)

S15 Table MR leave-one-out analysis of the causal effect of years of schooling on smoking. (XLSX)

S16 Table MR leave-one-out analysis of the causal effect of years of schooling on income. (XLSX)

S17 Table MR leave-one-out analysis of the causal effect of neuroticism on major depressive disorder. (XLSX)

S18 Table MR leave-one-out analysis of the causal effect of body mass index on major depressive disorder. (XLSX)

S19 Table MR leave-one-out analysis of the causal effect of smoking on major depressive disorder. (XLSX)

S20 Table MR leave-one-out analysis of the causal effect of income on major depressive disorder. (XLSX)

## List of abbreviations

MDD: major depressive disorder
BMI: body mass index
Education: years of schooling
Income: average total household income before tax
OR: odds ratio
CI: confidence intervals
PM: proportion mediated
SNP: single-nucleotide polymorphism
MR: Mendelian randomization
GWAS: genome-wide association study
IVW: inverse-variance-weighted
MR-PRESSO: MR-pleiotropy residual sum outlier
MR-RAPS: MR-robust adjusted profile score

## References

[1] Abdoli N, Salari N, Darvishi N, Jafarpour S, Solaymani M, Mohammadi M, et al. The global prevalence of major depressive disorder (MDD) among the elderly: A systematic review and meta-analysis. Neurosci Biobehav Rev. 2022;132:1067–73. Epub 2021/11/08. doi: 10.1016/j.neubiorev.2021.10.041 . 34742925

[2] Zhdanava M, Pilon D, Ghelerter I, Chow W, Joshi K, Lefebvre P, et al. The Prevalence and National Burden of Treatment-Resistant Depression and Major Depressive Disorder in the United States. J Clin Psychiatry. 2021;82(2). Epub 2021/05/15. doi: 10.4088/JCP.20m13699 . 33989464

[3] Ferrari AJ, Somerville AJ, Baxter AJ, Norman R, Patten SB, Vos T, et al. Global variation in the prevalence and incidence of major depressive disorder: a systematic review of the epidemiological literature. Psychol Med. 2013;43(3):471–81. Epub 2012/07/27. doi: 10.1017/S0033291712001511 . 22831756

[4] Greenberg PE, Fournier AA, Sisitsky T, Simes M, Berman R, Koenigsberg SH, et al. The Economic Burden of Adults with Major Depressive Disorder in the United States (2010 and 2018). Pharmacoeconomics. 2021;39(6):653–65. Epub 2021/05/06. doi: 10.1007/s40273-021-01019-4 ; PubMed Central PMCID: PMC8097130. 33950419 PMC8097130

[5] Hong C, Liu Z, Gao L, Jin Y, Shi J, Liang R, et al. Global trends and regional differences in the burden of anxiety disorders and major depressive disorder attributed to bullying victimisation in 204 countries and territories, 1999–2019: an analysis of the Global Burden of Disease Study. Epidemiol Psychiatr Sci. 2022;31:e85. Epub 2022/11/29. doi: 10.1017/S2045796022000683 ; PubMed Central PMCID: PMC9714217. 36440549 PMC9714217

[6] Lu J, Xu X, Huang Y, Li T, Ma C, Xu G, et al. Prevalence of depressive disorders and treatment in China: a cross-sectional epidemiological study. Lancet Psychiatry. 2021;8(11):981–90. Epub 2021/09/25. doi: 10.1016/S2215-0366(21)00251-0 . 34559991

[7] Tuithof M, Ten Have M, van Dorsselaer S, Kleinjan M, Beekman A, de Graaf R. Course of subthreshold depression into a depressive disorder and its risk factors. J Affect Disord. 2018;241:206–15. Epub 2018/08/22. doi: 10.1016/j.jad.2018.08.010 . 30130686

[8] Daghlas I, Lane JM, Saxena R, Vetter C. Genetically Proxied Diurnal Preference, Sleep Timing, and Risk of Major Depressive Disorder. JAMA Psychiatry. 2021;78(8):903–10. Epub 2021/05/27. doi: 10.1001/jamapsychiatry.2021.0959 ; PubMed Central PMCID: PMC8156187. 34037671 PMC8156187

[9] Rice F, Sellers R, Hammerton G, Eyre O, Bevan-Jones R, Thapar AK, et al. Antecedents of New-Onset Major Depressive Disorder in Children and Adolescents at High Familial Risk. JAMA Psychiatry. 2017;74(2):153–60. Epub 2016/12/08. doi: 10.1001/jamapsychiatry.2016.3140 . 27926743

[10] Freeman A, Tyrovolas S, Koyanagi A, Chatterji S, Leonardi M, Ayuso-Mateos JL, et al. The role of socio-economic status in depression: results from the COURAGE (aging survey in Europe). BMC Public Health. 2016;16(1):1098. Epub 2016/10/21. doi: 10.1186/s12889-016-3638-0 ; PubMed Central PMCID: PMC5069819. 27760538 PMC5069819

[11] Raffetti E, Donato F, Forsell Y, Galanti MR. Longitudinal association between tobacco use and the onset of depressive symptoms among Swedish adolescents: the Kupol cohort study. Eur Child Adolesc Psychiatry. 2019;28(5):695–704. Epub 2018/10/14. doi: 10.1007/s00787-018-1237-6 ; PubMed Central PMCID: PMC6514114. 30315361 PMC6514114

[12] Rao WW, Zong QQ, Zhang JW, An FR, Jackson T, Ungvari GS, et al. Obesity increases the risk of depression in children and adolescents: Results from a systematic review and meta-analysis. J Affect Disord. 2020;267:78–85. Epub 2020/02/18. doi: 10.1016/j.jad.2020.01.154 . 32063576

[13] Thompson RJ, Kuppens P, Mata J, Jaeggi SM, Buschkuehl M, Jonides J, et al. Emotional clarity as a function of neuroticism and major depressive disorder. Emotion. 2015;15(5):615–24. Epub 2015/04/07. doi: 10.1037/emo0000067 ; PubMed Central PMCID: PMC4586306. 25844973 PMC4586306

[14] Emdin CA, Khera AV, Kathiresan S. Mendelian Randomization. JAMA. 2017;318(19):1925–6. Epub 2017/11/23. doi: 10.1001/jama.2017.17219 . 29164242

[15] Smith GD, Ebrahim S. ’Mendelian randomization’: can genetic epidemiology contribute to understanding environmental determinants of disease? Int J Epidemiol. 2003;32(1):1–22. Epub 2003/04/12. doi: 10.1093/ije/dyg070 . 12689998

[16] Carter AR, Sanderson E, Hammerton G, Richmond RC, Davey Smith G, Heron J, et al. Mendelian randomisation for mediation analysis: current methods and challenges for implementation. Eur J Epidemiol. 2021;36(5):465–78. Epub 2021/05/08. doi: 10.1007/s10654-021-00757-1 ; PubMed Central PMCID: PMC8159796. 33961203 PMC8159796

[17] Sanderson E. Multivariable Mendelian Randomization and Mediation. Cold Spring Harb Perspect Med. 2021;11(2). Epub 2020/04/29. doi: 10.1101/cshperspect.a038984 ; PubMed Central PMCID: PMC7849347. 32341063 PMC7849347

[18] Lee JJ, Wedow R, Okbay A, Kong E, Maghzian O, Zacher M, et al. Gene discovery and polygenic prediction from a genome-wide association study of educational attainment in 1.1 million individuals. Nat Genet. 2018;50(8):1112–21. Epub 2018/07/25. doi: 10.1038/s41588-018-0147-3 ; PubMed Central PMCID: PMC6393768. 30038396 PMC6393768

[19] Luciano M, Hagenaars SP, Davies G, Hill WD, Clarke TK, Shirali M, et al. Association analysis in over 329,000 individuals identifies 116 independent variants influencing neuroticism. Nat Genet. 2018;50(1):6–11. Epub 2017/12/20. doi: 10.1038/s41588-017-0013-8 ; PubMed Central PMCID: PMC5985926. 29255261 PMC5985926

[20] Wray NR, Ripke S, Mattheisen M, Trzaskowski M, Byrne EM, Abdellaoui A, et al. Genome-wide association analyses identify 44 risk variants and refine the genetic architecture of major depression. Nat Genet. 2018;50(5):668–81. Epub 2018/04/28. doi: 10.1038/s41588-018-0090-3 ; PubMed Central PMCID: PMC5934326. 29700475 PMC5934326

[21] Pierce BL, Ahsan H, Vanderweele TJ. Power and instrument strength requirements for Mendelian randomization studies using multiple genetic variants. Int J Epidemiol. 2011;40(3):740–52. Epub 2010/09/04. doi: 10.1093/ije/dyq151 ; PubMed Central PMCID: PMC3147064. 20813862 PMC3147064

[22] Davies NM, Holmes MV, Davey Smith G. Reading Mendelian randomisation studies: a guide, glossary, and checklist for clinicians. BMJ. 2018;362:k601. Epub 2018/07/14. doi: 10.1136/bmj.k601 ; PubMed Central PMCID: PMC6041728 interests and declare that we have no competing interests. 30002074 PMC6041728

[23] Bowden J, Davey Smith G, Burgess S. Mendelian randomization with invalid instruments: effect estimation and bias detection through Egger regression. Int J Epidemiol. 2015;44(2):512–25. Epub 2015/06/08. doi: 10.1093/ije/dyv080 ; PubMed Central PMCID: PMC4469799. 26050253 PMC4469799

[24] Xue H, Shen X, Pan W. Constrained maximum likelihood-based Mendelian randomization robust to both correlated and uncorrelated pleiotropic effects. Am J Hum Genet. 2021;108(7):1251–69. Epub 2021/07/03. doi: 10.1016/j.ajhg.2021.05.014 ; PubMed Central PMCID: PMC8322939. 34214446 PMC8322939

[25] Verbanck M, Chen CY, Neale B, Do R. Detection of widespread horizontal pleiotropy in causal relationships inferred from Mendelian randomization between complex traits and diseases. Nat Genet. 2018;50(5):693–8. Epub 2018/04/25. doi: 10.1038/s41588-018-0099-7 ; PubMed Central PMCID: PMC6083837. 29686387 PMC6083837

[26] Zhao Q, Wang J, Hemani G, Bowden J, Small DSJTAoS. Statistical inference in two-sample summary-data Mendelian randomization using robust adjusted profile score. 2020;48(3):1742–69. doi: 10.1214/19-AOS1866

[27] Brion MJ, Shakhbazov K, Visscher PM. Calculating statistical power in Mendelian randomization studies. Int J Epidemiol. 2013;42(5):1497–501. Epub 2013/10/26. doi: 10.1093/ije/dyt179 ; PubMed Central PMCID: PMC3807619. 24159078 PMC3807619

[28] Hemani G, Zheng J, Elsworth B, Wade KH, Haberland V, Baird D, et al. The MR-Base platform supports systematic causal inference across the human phenome. Elife. 2018;7. Epub 2018/05/31. doi: 10.7554/eLife.34408 ; PubMed Central PMCID: PMC5976434. 29846171 PMC5976434

[29] Julian TH, Glascow N, Barry ADF, Moll T, Harvey C, Klimentidis YC, et al. Physical exercise is a risk factor for amyotrophic lateral sclerosis: Convergent evidence from Mendelian randomisation, transcriptomics and risk genotypes. EBioMedicine. 2021;68:103397. Epub 2021/05/30. doi: 10.1016/j.ebiom.2021.103397 ; PubMed Central PMCID: PMC8170114. 34051439 PMC8170114

[30] Li R, Chen Y, Zhao A, Huang L, Long Z, Kang W, et al. Exploring genetic association of insomnia with allergic disease and asthma: a bidirectional Mendelian randomization study. Respir Res. 2022;23(1):84. Epub 2022/04/09. doi: 10.1186/s12931-022-02009-6 ; PubMed Central PMCID: PMC8991606. 35392909 PMC8991606

[31] Lewis RG, Simpson B. Genetics, Autosomal Dominant. In: Lewis RG, Simpson B, editors. StatPearls. Treasure Island (FL) 2022.

[32] Qi L. Mendelian randomization in nutritional epidemiology. Nutr Rev. 2009;67(8):439–50. Epub 2009/08/14. doi: 10.1111/j.1753-4887.2009.00218.x ; PubMed Central PMCID: PMC3671930. 19674341 PMC3671930

[33] Liu Q, He H, Yang J, Feng X, Zhao F, Lyu J. Changes in the global burden of depression from 1990 to 2017: Findings from the Global Burden of Disease study. J Psychiatr Res. 2020;126:134–40. Epub 2019/08/24. doi: 10.1016/j.jpsychires.2019.08.002 . 31439359

[34] Kranjac AW, Nie J, Trevisan M, Freudenheim JL. Depression and body mass index, differences by education: Evidence from a population-based study of adult women in the U.S. Buffalo-Niagara region. Obes Res Clin Pract. 2017;11(1):63–71. Epub 2016/03/31. doi: 10.1016/j.orcp.2016.03.002 ; PubMed Central PMCID: PMC5035174. 27025915 PMC5035174

[35] Wickersham A, Ford T, Stewart R, Downs J. Estimating the impact of child and early adolescent depression on subsequent educational attainment: secondary analysis of an existing data linkage. Epidemiol Psychiatr Sci. 2021;30:e76. Epub 2022/05/04. doi: 10.1017/S2045796021000603 ; PubMed Central PMCID: PMC8679834. 35502824 PMC8679834

[36] Hill WD, Weiss A, Liewald DC, Davies G, Porteous DJ, Hayward C, et al. Genetic contributions to two special factors of neuroticism are associated with affluence, higher intelligence, better health, and longer life. Mol Psychiatry. 2020;25(11):3034–52. Epub 2019/03/15. doi: 10.1038/s41380-019-0387-3 ; PubMed Central PMCID: PMC7577854. 30867560 PMC7577854

[37] Widiger TA, Oltmanns JR. Neuroticism is a fundamental domain of personality with enormous public health implications. World Psychiatry. 2017;16(2):144–5. Epub 2017/05/13. doi: 10.1002/wps.20411 ; PubMed Central PMCID: PMC5428182. 28498583 PMC5428182

[38] Bondy E, Baranger DAA, Balbona J, Sputo K, Paul SE, Oltmanns TF, et al. Neuroticism and reward-related ventral striatum activity: Probing vulnerability to stress-related depression. J Abnorm Psychol. 2021;130(3):223–35. Epub 2021/02/05. doi: 10.1037/abn0000618 ; PubMed Central PMCID: PMC8110089. 33539118 PMC8110089

[39] Wongpakaran N, Wongpakaran T, van Reekum R. Social inhibition as a mediator of neuroticism and depression in the elderly. BMC Geriatr. 2012;12:41. Epub 2012/08/04. doi: 10.1186/1471-2318-12-41 ; PubMed Central PMCID: PMC3445846. 22856615 PMC3445846

[40] Speed D, Hemani G, Speed MS, Major Depressive Disorder Working Group of the Psychiatric Genomics C, Borglum AD, Ostergaard SD. Investigating the causal relationship between neuroticism and depression via Mendelian randomization. Acta Psychiatr Scand. 2019;139(4):395–7. Epub 2019/01/31. doi: 10.1111/acps.13009 ; PubMed Central PMCID: PMC6426667. 30697695 PMC6426667

[41] Jaconelli A, Stephan Y, Canada B, Chapman BP. Personality and physical functioning among older adults: the moderating role of education. J Gerontol B Psychol Sci Soc Sci. 2013;68(4):553–7. Epub 2012/10/17. doi: 10.1093/geronb/gbs094 ; PubMed Central PMCID: PMC3859357. 23070900 PMC3859357

[42] Luppino FS, de Wit LM, Bouvy PF, Stijnen T, Cuijpers P, Penninx BW, et al. Overweight, obesity, and depression: a systematic review and meta-analysis of longitudinal studies. Arch Gen Psychiatry. 2010;67(3):220–9. Epub 2010/03/03. doi: 10.1001/archgenpsychiatry.2010.2 . 20194822

[43] Mannan M, Mamun A, Doi S, Clavarino A. Is there a bi-directional relationship between depression and obesity among adult men and women? Systematic review and bias-adjusted meta analysis. Asian J Psychiatr. 2016;21:51–66. Epub 2016/05/22. doi: 10.1016/j.ajp.2015.12.008 . 27208458

[44] Li Y, Cai T, Wang H, Guo G. Achieved educational attainment, inherited genetic endowment for education, and obesity. Biodemography Soc Biol. 2021;66(2):132–44. Epub 2021/06/30. doi: 10.1080/19485565.2020.1869919 ; PubMed Central PMCID: PMC8607810. 34182851 PMC8607810

[45] Mosli HH, Kutbi HA, Alhasan AH, Mosli RH. Understanding the Interrelationship between Education, Income, and Obesity among Adults in Saudi Arabia. Obes Facts. 2020;13(1):77–85. Epub 2020/01/20. doi: 10.1159/000505246 ; PubMed Central PMCID: PMC7098293. 31955158 PMC7098293

[46] Hecht SS. Cigarette smoking: cancer risks, carcinogens, and mechanisms. Langenbecks Arch Surg. 2006;391(6):603–13. Epub 2006/10/13. doi: 10.1007/s00423-006-0111-z . 17031696

[47] Fluharty M, Taylor AE, Grabski M, Munafo MR. The Association of Cigarette Smoking With Depression and Anxiety: A Systematic Review. Nicotine Tob Res. 2017;19(1):3–13. Epub 2016/05/21. doi: 10.1093/ntr/ntw140 ; PubMed Central PMCID: PMC5157710. 27199385 PMC5157710

[48] Watanapongvanich S, Khan MSR, Putthinun P, Ono S, Kadoya Y. Financial Literacy, Financial Education, and Smoking Behavior: Evidence From Japan. Front Public Health. 2020;8:612976. Epub 2021/02/02. doi: 10.3389/fpubh.2020.612976 ; PubMed Central PMCID: PMC7844398. 33520921 PMC7844398

[49] Hoebel J, Kuntz B, Kroll LE, Finger JD, Zeiher J, Lange C, et al. Trends in Absolute and Relative Educational Inequalities in Adult Smoking Since the Early 2000s: The Case of Germany. Nicotine Tob Res. 2018;20(3):295–302. Epub 2017/04/22. doi: 10.1093/ntr/ntx087 . 28431153

[50] Setter C, Peter R, Siegrist J, Hort W. Impact of school and vocational education on smoking behaviour: results from a large-scale study on adolescents and young adults in Germany. Soz Praventivmed. 1998;43(3):133–40. Epub 1998/08/11. doi: 10.1007/BF01359721 . 9697252

[51] Hinata A, Kabasawa K, Watanabe Y, Kitamura K, Ito Y, Takachi R, et al. Education, household income, and depressive symptoms in middle-aged and older Japanese adults. BMC Public Health. 2021;21(1):2120. Epub 2021/11/20. doi: 10.1186/s12889-021-12168-8 ; PubMed Central PMCID: PMC8600755. 34794416 PMC8600755

[52] Korhonen K, Remes H, Martikainen P. Education as a social pathway from parental socioeconomic position to depression in late adolescence and early adulthood: a Finnish population-based register study. Soc Psychiatry Psychiatr Epidemiol. 2017;52(1):105–16. Epub 2016/10/21. doi: 10.1007/s00127-016-1296-2 . 27761600

[53] Solomon BC, Nikolaev BN, Shepherd DA. Does educational attainment promote job satisfaction? The bittersweet trade-offs between job resources, demands, and stress. J Appl Psychol. 2022;107(7):1227–41. Epub 2022/06/24. doi: 10.1037/apl0000904 . 35737558

[54] Lynch SM. Explaining life course and cohort variation in the relationship between education and health: the role of income. J Health Soc Behav. 2006;47(4):324–38. Epub 2007/01/24. doi: 10.1177/002214650604700402 . 17240923

