# Supplementary figures and images for: Four modifiable factors that mediate the effect of educational time on major depressive disorder risk: A network Mendelian randomization study
Source: PLoS One. 2023 Jul 12;18(7):e0288034. doi: 10.1371/journal.pone.0288034 (PMC10337944; doi:10.1371/journal.pone.0288034)

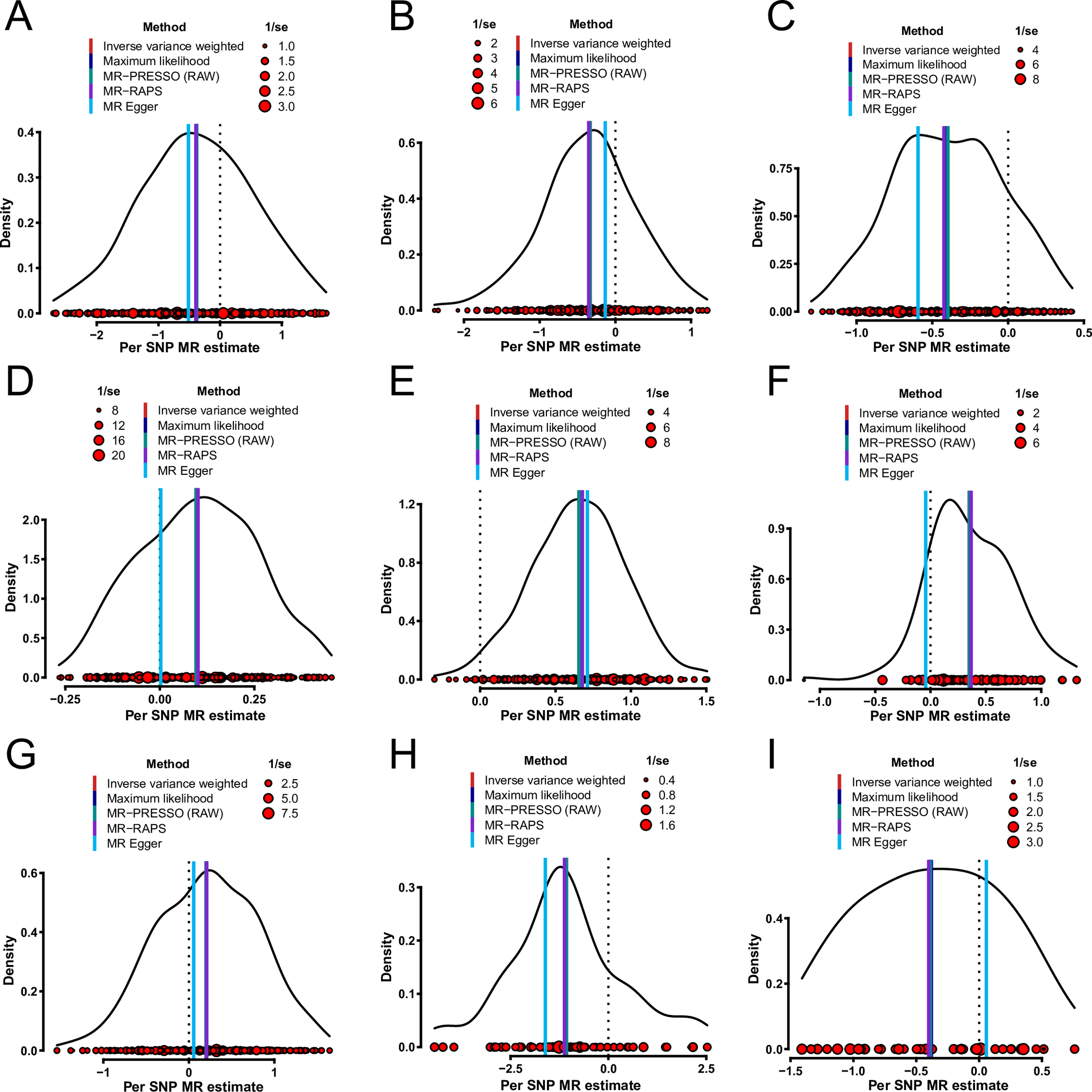

Supplement: S1 Fig — (A) Effect of education-related SNPs on the risk of MDD. (B) Effect of education-related SNPs on neuroticism. (C) Effect of education-related SNPs on BMI. (D) Effect of education-related SNPs on smoking. (E) Effect of education-related SNPs on income. (F) Effect of neuroticism-related SNPs on the risk of MDD. (G) Effect of BMI-related SNPs on the risk of MDD. (H) Effect of smoking-related SNPs on the risk of MDD. (I) Effect of income-related SNPs on the risk of MDD. MDD = major depressive disorder; BMI = body mass index; Education = years of schooling; Smoking = smoking (ever vs. never); Income = average total household income before tax; MR = Mendelian randomization; SNP = single nucleotide polymorphism; IVW = inverse-variance-weighted; MR-PRESSO = MR-pleiotropy residual sum outlier; MR-RAPS = MR-robust adjusted profile score. (TIF) [file pone.0288034.s001.tif]
